# Basilar Stenosis Reduces the Impact of Successful Recanalization on Outcome in Basilar Artery Occlusion

**DOI:** 10.3390/diagnostics14212348

**Published:** 2024-10-22

**Authors:** Moritz R. Hernandez Petzsche, Philip Hoelter, Sebastian Rühling, Julian Schwarting, Benno Ikenberg, Silke Wunderlich, Christian Maegerlein, Claus Zimmer, Maria Berndt-Mück, Tobias Boeckh-Behrens

**Affiliations:** 1 Department of Diagnostic and Interventional Neuroradiology, Klinikum rechts der Isar, School of Medicine and Health, Technical University of Munich, 81675 Munich, Germany; sebastian.ruehling@tum.de (S.R.); julian.schwarting@tum.de (J.S.); christian.maegerlein@tum.de (C.M.); claus.zimmer@tum.de (C.Z.); maria.berndt@tum.de (M.B.-M.); boeckh-behrens@tum.de (T.B.-B.); 2 Department of Neuroradiology, Universitätsklinikum Erlangen, 91054 Erlangen, Germany; philip.hoelter@kliniken-sob.de; 3 Department of Neurology, Klinikum rechts der Isar, School of Medicine and Health, Technical University of Munich, 81675 Munich, Germany; benno.ikenberg@tum.de (B.I.); silke.wunderlich@tum.de (S.W.)

**Keywords:** stroke, basilar, occlusion, TICI, outcome, thrombus, embolism, stenosis

## Abstract

Background: Evidence from randomized controlled trials has shown a benefit for endovascular treatment in basilar artery occlusion. We aimed to show the effect of the recanalization result on outcome and determine the role of underlying basilar stenosis in a real-world setting. Methods: A retrospective, single-center study of patients who received endovascular treatment for basilar artery occlusion from March 2008 to June 2022 was conducted. Clinical and outcome characteristics were gathered. Multivariate logistic regression analysis was performed to predict poor outcomes (post-treatment mRS 5 or 6). MRS shift analysis was performed. Results: This study includes 210 patients (mean age, 71.4 years +/− 13.3 [standard deviation]; 124 men). The variables age (OR, 1.04; 95% CI: 1.01–1.08; *p* = 0.014), underlying basilar stenosis (OR: 4.86; 95% CI: 2.15–10.98; *p* < 0.001), admission NHISS (OR: 1.09; 95% CI: 1.04–1.13; *p* < 0.001), and TICI (OR: 1.89; 95% CI: 1.09–3.25; *p* = 0.022) independently predicted a poor outcome. Patients with occlusions due to underlying stenosis had significantly worse recanalization rates. Median post-treatment mRS in all patients with embolic occlusion was 4; IQR, 2–5 (only patients with embolic occlusion: mTICI 0-2a, median: 5 [IQR, 4–5.5]; mTICI 2b, median: 4 [IQR, 2.5–6]; mTICI 3, median: 3 [IQR, 1–5]; *p* = 0.037). Median post-treatment mRS in all patients with occlusions due to underlying stenosis was 5; IQR, 4–6 (only patients with embolic occlusions: mTICI 0-2a, median: 6 [IQR, 4.5–6]; mTICI 2b, median: 6 [IQR, 4.25–6]; mTICI 3, median: 5 [IQR, 3.5–5.25]; *p* = 0.059). Conclusions: Successful recanalization is essential for preventing poor outcomes in basilar artery occlusion. Underlying basilar stenosis diminishes the effect of successful recanalization on the overall outcome.

## 1. Introduction

Basilar artery occlusion (BAO) is a rare form of stroke, responsible for about 1% of all cerebral ischemias and about 5 to 10% of all large vessel occlusions [1,2]. Due to the eloquent nature of basilar artery (BA)-dependent brain tissue, including the brain stem, BAO often leads to devastating outcomes. Despite the best medical treatment, up to 80% of patients with BAO remain severely disabled or die [3,4,5]. Revascularization treatment of BAO includes both intravenous thrombolysis (IVT) and endovascular therapy (EVT). The effectiveness of EVT for BAO has been recently confirmed in two randomized-controlled trials (RCTs), with both trials demonstrating significantly better outcomes with EVT performed up to 24 h after stroke onset [4,6].

BAO is caused by clot embolism or by in situ thrombosis due to plaque rupture in patients with underlying basilar stenosis (BS). BAO due to BS requires more complex interventional therapy, usually including stenting, and has been shown in recent studies to be associated with a worse outcome than embolic BAO [7,8,9,10]. In previous studies, including recent RCTs, the varying etiologies of BAO and their impact on patient prognosis and treatment outcomes were not specifically addressed [4,6].

In this large single-center cohort, we aimed to assess the influence of reperfusion success on clinical outcomes and to determine the effect of different etiologies of BAO on treatment success.

## 2. Materials and Methods

### 2.1. Study Population

This retrospective, single-center study included all consecutive patients who were admitted for ischemic stroke due to acute BAO and treated with EVT at our comprehensive stroke center between March 2008 and June 2022 (*n* = 210). There were no exclusion criteria. Part of the patient cohort has been previously described [8,9,11,12]. IVT was administered to some patients before intervention based on national and center-specific stroke treatment guidelines.

### 2.2. Ethical Approval

Approval was obtained by the local ethics board in accordance with regional law under reference number 274/21 S-SR. Patient informed consent was waived by the ethics committee due to the retrospective nature of the study.

### 2.3. Clinical, Procedural, and Outcome Parameters

Patients’ clinical, demographic, procedural, and outcome data were acquired retrospectively. National Institutes of Health Stroke Scale (NIHSS) was assessed by neurologists at the time of admission and discharge as part of the clinical routine. Substantial neurological improvement was defined as the difference between admission and discharge NIHSS score of ≤8 or discharge NIHSS score of ≤1, as previously described [8,11,13,14]. MRS score was used to measure disability before stroke onset, at admission, at discharge (referred to as post-treatment in the text below), and after 3 months. A poor clinical outcome, used as endpoint for regression analysis, was defined as post-treatment mRS score of 5 (bedridden with severe disability at the time of discharge) or 6 (death during the course of hospital stay), as described previously [15]. A good clinical outcome was defined as post-treatment mRS between 0 and 3, as described previously [8,11]. As there was limited availability of mRS scores after 3 months, this variable was unsuitable as a regression endpoint. All patients underwent follow-up brain imaging within 24h after intervention, which was used to diagnose potential bleeding complications. Stroke etiology was classified according to the Trial of Org 10,172 in Acute Stroke Treatment (TOAST) criteria [16]. Reperfusion success of endovascular therapy was quantified based on the modified Thrombolysis in Cerebral Infarction (mTICI) scale by two experienced neuroradiologists [17].

### 2.4. Statistical Analysis

Variables with metric and ordinal data were described as median and interquartile range (IQR). Categorical variables were described using absolute frequencies and percentages.

The Mann–Whitney U test was employed for univariate analysis when comparing two groups with metric or ordinal data. This test assumes that the distributions of the groups are similarly shaped, which was confirmed by the data in our analysis. For comparisons involving three or more groups with metric or ordinal data, the Kruskal–Wallis test was used. Both tests assume independence of the data, which was satisfied in this study. These non-parametric methods, chosen because they do not assume normal distribution, ensured robust analysis, as some data may not have met the normality criteria for parametric tests. The Pearson Chi-Square test was applied to compare categorical variables of three or more groups. Fisher’s exact test was used for dichotomous categorical variables. *p*-values below 0.05 were considered statistically significant.

Following univariate analysis, a multivariate logistic regression model using a stepwise forward variable selection method was performed to determine variables predicting a poor outcome. Statistical analyses were carried out using SPSS 26 (IBM Corporation, Armonk, NY, USA).

## 3. Results

### 3.1. Baseline Patient Characteristics

This study includes 210 patients treated with EVT for BAO at our center between 2008 and 2022 (median age: 74 years [IQR, 63–81], 124 men). The patients included in this study were split into three groups based on angiographic outcome as measured by mTICI. In 137 (65.2%) patients, complete recanalization (mTICI 3) was achieved, while 50 (23.8%) had successful reperfusion of half or greater of the downstream ischemic territory (mTICI 2b). Recanalization failed in 23 (11%) of all patients (<TICI 2b). No significant differences were observed between these groups regarding age or gender distribution. The most common cardiovascular risk factor was arterial hypertension, occurring in 159 cases (71.8%), followed by atrial fibrillation in 81 cases (39.1%). No significant differences were found in the rates of occurrence of any cardiovascular risk factor between the groups. There was low pre-stroke disability in the cohort (median mRS = 0, IQR 0–0.5). Median admission NHISS scores were 13 (IQR 6.5–22). The most common etiology of stroke was cardioembolism (80 patients, 39.0%), closely followed by large-artery arteriosclerosis (67 patients, 32.7%). Patients with lower TICI scores were more likely to have large-artery arteriosclerosis as a stroke etiology (*p* = 0.02). Correspondingly and more specifically, there were significantly more patients with BS as etiology of BAO among the groups with lower angiographic outcome (*p* = 0.04). Conversely, the rate of successful recanalization was higher among patients with BAO due to BS, as has been demonstrated on the part of the same cohort [9]; see Figure S1. Other stroke etiologies based on the TOAST classification occurred at a similar rate between groups. The median time between symptom onset and groin puncture was 265 min (IQR, 186–385). IVT was administered in 84 patients (40%). IVT did not have a significant impact on patient outcome (median post-treatment mRS of patients that received IVT = 4 [IQR 2–5.5] and median post-treatment mRS of patients that did not receive IVT = 4.5 [IQR 2–6]; *p* = 0.269). Table 1 provides an overview of the baseline clinical and admission characteristics.

### 3.2. Endovascular Treatment, Clinical, and Safety Outcomes

EVT was performed under primary general anesthesia in 98% of cases (100 patients; data regarding anesthesia care was missing for 108 cases). Only one case was performed in conscious sedation and one other with primary conscious sedation and secondary general anesthesia (1%, respectively). The intervention lasted a median of 60 min (IQR 31–105). Patients with better angiographic outcomes had significantly shorter procedure times (mTICI 0-2a, median: 83 min [IQR, 50–146]; mTICI 2b, median: 80 min [IQR, 48–124.5]; mTICI 3, median: 45 min [IQR, 25.5–95]; *p* < 0.001). For all patients, there were a median of two reperfusion attempts (IQR, 1–3). Basilar stenting was performed in 47 patients (22.4%), and stenting of the vertebral artery was performed in 17 patients (8.1%).

Median post-treatment NIHSS was 10 (IQR 2-42); as expected, better angiographic outcomes lead to lower post-treatment NIHSS scores (mTICI 0-2a, median: 25 [IQR, 5–42]; mTICI 2b, median: 11 [IQR, 4–42]; mTICI 3, median: 7.5 [IQR, 2–17.75]; *p* = 0.005). Similarly, patients with better TICI scores were more likely to have a substantial neurological improvement (mTICI 0-2a, 4 patients [19%]; mTICI 2b, 10 [21.3%]; mTICI 3, 55 [43.0%]; *p* = 0.008). Median post-treatment mRS was 4 (IQR, 2–6) for all patients. Post-treatment disability was significantly higher in patients with worse TICI scores (mTICI 0-2a, median: 5 [IQR, 4–6]; mTICI 2b, median: 5 [IQR, 3–6]; mTICI 3, median: 4 [IQR, 1–5]; *p* = 0.001), and a good clinical outcome (mRS 0-3) was significantly more likely with better angiographic outcome. Data availability for mRS scores after three months was limited (125 cases with missing); however, the same trend as with post-treatment mRS was evident without reaching statistical significance (mTICI 0-2a, median: 6 [IQR, 3–6]; mTICI 2b, median: 4 [IQR, 1–6]; mTICI 3, median: 3 [IQR, 1–6]; *p* = 0.09). Symptomatic ICH occurred only in 6 patients, all of which were in the TICI 3 group; no statistical significance was found for its rate of occurrence. In-house mortality occurred in 50 patients (24.9%) and was more likely in patients with worse angiographic outcomes (*p* = 0.008). Table 2 shows an overview of all procedural, outcome, and safety characteristics.

### 3.3. Predictors of Poor Outcome

In univariate analysis, the variable ages, underlying BS, admission NIHSS, diabetes mellitus, angiographic outcome as measured by mTICI, reperfusion attempts, interventional duration, sole aspiration, and basilar stenting were significantly associated with a poor outcome, defined as a post-treatment mRS, of 5 or 6. An overview of the univariate analysis can be found in Table S1 of the supplement. All variables significantly correlated to poor outcomes in univariate analysis were entered into a multivariate logistic regression model using a stepwise forward variable selection process. The multivariate regression model returned the variables age (odds ratio [OR], 1.04; 95% CI: 1.01–1.08; *p* = 0.014), underlying BS (OR: 4.86; 95% CI: 2.15–10.98; *p* < 0.001), admission NHISS (OR: 1.09; 95% CI: 1.04–1.13; *p* < 0.001), and TICI (OR: 1.89; 95% CI: 1.09–3.25; *p* = 0.022) as independent predictors of a poor outcome. Table 3 summarizes the findings of the regression analysis. Figure S2 shows a forest plot displaying the regression results.

### 3.4. The Effect of Underlying Stenosis

**Figure 1:**
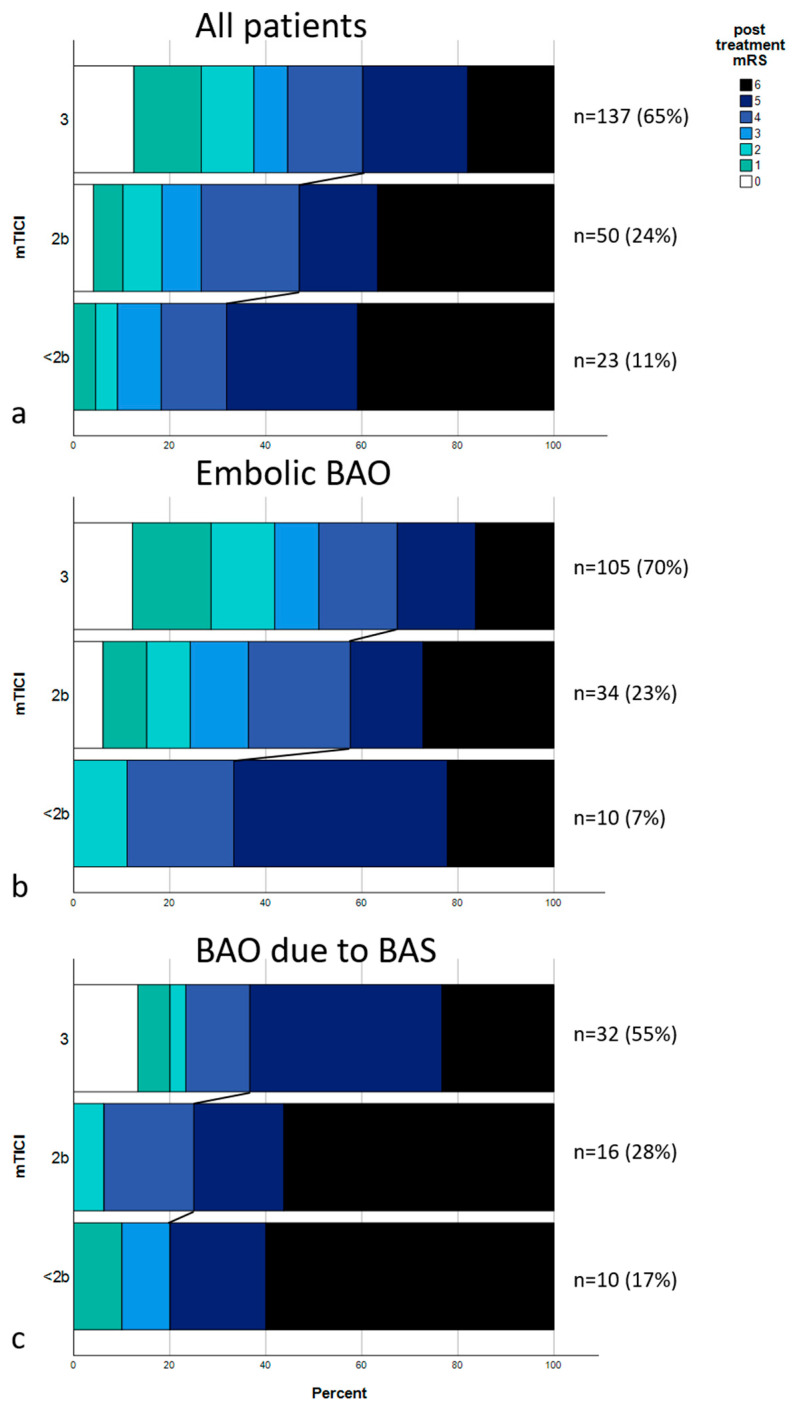
Distribution of post-treatment mRS scores. (**a**) mRS scores of the entire cohort divided upon recanalization results (mTICI 3, mTICI 2b, and mTICI < 2b). Connecting lines show the mRS shift between the groups, dividing patients with a poor outcome (mRS 5-6) from the rest. (**b**) mRS distribution of all embolic occlusions. (**c**) mRS distributions of all occlusions due to underlying stenosis.

**Figure 2:**
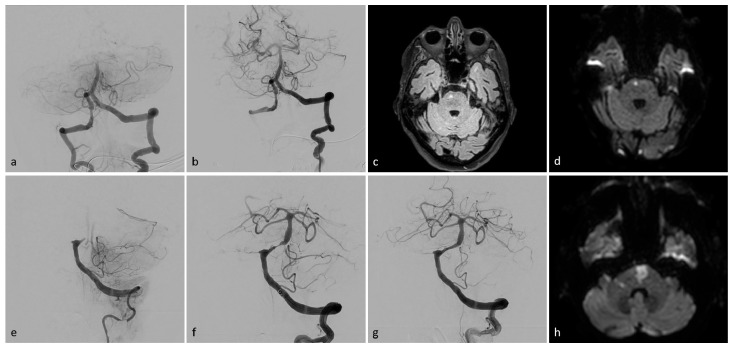
Exemplary patient imaging. Intraprocedural angiographic images (**a**,**b**,**e**–**g**), fluid-attenuated inversion recovery (FLAIR), magnetic resonance imaging (MRI, (**c**)), and diffusion-weighted images (DWI, (**d**,**h**)). Patient I (**a**–**d**): A 73-year-old man presented with left-sided weakness and vertigo, followed by a coma. Initial angiography revealed occlusion of the basilar head (**a**). Successful TICI 3 recanalization was achieved after a single aspiration thrombectomy (**b**). MR-imaging showed a small paramedian pontine infarct on the right side (**c**,**d**). The patient was discharged with mild left-sided hemiparesis (mRS 2). Patient II (**e**–**h**): A 78-year-old woman presented with acute-onset tetraplegia. Initial angiography (**e**) showed proximal occlusion of the basilar artery. Post-thrombectomy, high-grade stenosis was observed in the proximal third of the basilar artery (**f**). After percutaneous transluminal angioplasty (PTA) and stenting, the stenosis resolved, with a final result of TICI 3 (**g**). DW imaging revealed an infarct in the pons and a small infarct in the right cerebellar peduncle (**h**). The patient was discharged with persistent tetraparesis, unable to walk without assistance (mRS 4).

The multivariate analysis raises the question if the effect on outcome achieved by recanalization success is impeded by an underlying stenosis. Median post-treatment mRS in all patients with embolic BAO was 4; IQR, 2–5 (only patients with embolic BAO: mTICI 0-2a, median: 5 [IQR, 4–5.5]; mTICI 2b, median: 4 [IQR, 2.5–6]; mTICI 3, median: 3 [IQR, 1–5]; *p* = 0.037). Median post-treatment mRS in all patients with BAO due to BS was 5; IQR, 4–6 (only patients with embolic BAO: mTICI 0-2a, median: 6 [IQR, 4.5–6]; mTICI 2b, median: 6 [IQR, 4.25–6]; mTICI 3, median: 5 [IQR, 3.5–5.25]; *p* = 0.059). A shift analysis was performed, as shown in Figure 1, showing distributions of post-treatment mRS scores for all patients (Figure 1a), for patients with embolic BAO (Figure 1b), and for patients with BAO due to BS (Figure 1c). Figure 2 shows two exemplary cases of BAO.

## 4. Discussion

This real-world, single-center study of 210 patients identifies successful recanalization as essential for preventing a poor outcome in patients with BAO, confirming the results of the two recently published randomized controlled trials that provide evidence for the efficacy of EVT in BAO [4,6]. Furthermore, confirming previous studies [7,8,10], underlying BS was an independent risk factor for poor outcomes. In this large single-center collective, we demonstrate that patients with BAO due to underlying BS have a lower chance of successful recanalization (lower mTICI scores) than their embolic counterparts. Patients with BAO due to BS profit less from successful recanalization than patients with embolic BAO. Furthermore, we demonstrate that the final outcome in BAO is closely dependent on recanalization success.

This study demonstrates that high rates of successful recanalization are feasible (89% mTICI ≥ 2b at our center). These recanalization rates are comparable to those reported in other studies [18,19]. Rates of poor outcome (death or mRS 5 in the post-treatment phase) were significantly reduced by better recanalization results, and low TICI scores were an independent predictor of poor outcome. However, even in patients with excellent recanalization results (TICI 3), rates of poor outcomes were at around 40%. Further factors are, therefore, relevant to the final outcome. We identified age, underlying BS, and admission NHISS as independent predictors of poor outcomes.

High-admission NHISS likely stands for larger areas of completed tissue infarction that cannot be saved by successful recanalization. Admission NHISS has been shown to be associated with worse outcomes both in the anterior and posterior circulation [19,20]. Age, an indicator of overall patient frailty, has also been shown in previous studies to be associated with a poor outcome after stroke [21,22].

Underlying BS has been previously identified as a risk factor for worse outcomes in BAO [7,8,9,10]. Stenotic occlusions of the basilar artery are located more proximally in the basilar artery and usually require more complex interventional treatment, including stenting, to prevent short-term re-occlusion. Worse angiographic outcomes in patients with stenotic occlusions in comparison to their embolic counterparts have been previously shown on part of this same cohort [9] and in other studies [23]. Previous studies have developed methods of identifying stenotic occlusions on CTA, facilitating interventional planning and workflow [8,12]. MRS shift analysis performed in this study shows poor overall outcomes in patients with BAO due to BS. Successful recanalization improved outcomes for these patients but less so than in their embolic counterparts. Median outcomes were not significantly improved by successful recanalization in patients with underlying BS; however, this is likely partially due to the smaller group size (*n* = 58). These findings highlight a potential unmet need for this sub-group of BAO to further develop interventional material and techniques tailored to this occlusion type.

Forty years after the first recorded catheter-based treatment of BAO [24], the year 2022 saw two RCTs that produced high-level evidence for EVT in BAO [4,6]. This study adds to a large body of evidence, largely stemming from retrospective or prospective registry data, showing a positive effect of EVT in BAO [5,19,25,26,27,28,29,30,31,32,33] and underlines the importance of achieving the best possible recanalization result in preventing poor outcomes. In this study, we highlight underlying BS as a complicating factor, which diminishes the return of successful recanalization on patient outcomes. In planning future randomized controlled trials, this subgroup should be given consideration as a factor with the potential to reduce the positive effect on outcomes generated by EVT.

### Limitations

This study is limited by its retrospective design. Furthermore, the most readily available outcome scores are collected during in-house stays for stroke patients. MRS scores at 90 days, most frequently used to evaluate stroke outcomes, were not available for statistical analysis in over half the cohort. However, mRS scores at discharge have also been frequently used as a measure of outcome in the past [34,35]. This study is limited by its lack of study lacks a control group without EVT and, therefore, only measures the effect of reperfusion success in patients undergoing endovascular treatment. Furthermore, the TICI score has been criticized previously for the difficulty in adequately classifying residual side branch occlusion [11,36]. Alternative scales to measure posterior circulation recanalization success have been introduced [11]. However, these alternative scores have not been widely established in clinical practice, and the TICI score remains the current standard for grading reperfusion success in posterior circulation stroke, including in RCTs [6,37]. The extensive timeframe covered in this study presents another limitation: advancements in procedures and technology may lead to improved angiographic outcomes, while conversely, the expansion of thrombectomy indications to later time windows after stroke onset could negatively affect outcomes in more recent cases. It is important to note that no analyses were conducted in this context, as we believe this question does not hinder the primary focus of the manuscript. Additionally, data on coronary artery disease and other atherosclerotic conditions—important cardiovascular risk factors and potentially linked to intracranial atherosclerosis, such as BS—were not collected in this study. Consequently, we are unable to evaluate the potential impact of peripheral atherosclerotic disease on angiographic and clinical outcomes as well as on the occurrence of BS.

## 5. Conclusions

Recanalization success is essential for preventing a poor outcome in BAO and should be pursued aggressively. Patients with underlying BS are less likely to have successful recanalization. In turn, patients with underlying BS profit less from successful intervention and have overall poor outcomes. This patient group should be given special consideration in the design of future studies as they may play a role in diminishing outcome effects achieved by EVT. Development of new techniques for EVT in BAO with underlying stenosis may be required to improve overall outcomes in these patients.

## Figures and Tables

**Table 1:** Baseline characteristics.

Baseline Characteristics	All Patients	mTICI 0-2a	mTICI 2b	mTICI 3	*p*=
*n* (%)	210	23 (11.0)	50 (23.8)	137 (65.2)	
Age, median (IQR)	74 (63–81)	75 (69–80)	74 (63–81)	74 (60–82)	0.73
Male, *n* (%)	124 (59.0)	14 (60.9)	32 (64.0)	124 (56.9)	0.67
Cardiovascular risk factors, *n* (%)					
atrial fibrillation	81 (39.1)	6 (26.1)	23 (47.9)	52 (38.2)	0.2
arterial hypertension	150 (71.8)	20 (87.0)	31 (62.0)	99 (72.8)	0.08
diabetes mellitus	33 (16.1)	5 (21.7)	7 (14.6)	21 (15.7)	0.73
Dyslipidemia	44 (21.0)	8 (34.8)	7 (14.0)	29 (21.2)	0.13
previous stroke or TIA	47 (22.7)	4 (17.4)	11 (22.4)	32 (23.7)	0.8
Pre-stroke mRS, median (IQR), (129 missing)	0 (0.0–0.5)	0 (0.0–1.0)	0 (0.0–0.5)	0 (0.0–1.0)	0.92
Admission NIHSS, median (IQR), (25 missing)	13 (6.5–22)	11.5 (4.0–23.25)	13 (6.5–22.0)	14 (7.0–22.0)	0.84
TOAST classification, *n* (%), (5 missing)					
Large-artery atherosclerosis	67 (32.7)	13 (59.1)	17 (34.0)	37 (27.8)	0.018
Cardioembolism	80 (39.0)	5 (22.7)	21 (42.0)	54 (40.6)	0.22
Small-artery occlusion	NA	NA	NA	NA	NA
Other determined etiology	14 (6.8)	0 (0.0)	4 (8.0)	10 (7.5)	0.39
Undetermined etiology	44 (21.5)	4 (18.2)	8 (16.0)	32 (7.5)	0.5
Underlying basilar stenosis *n* (%), (3 missing)	58 (28.0)	10 (50.0)	16 (32.0)	32 (23.4)	0.036
Onset to groin puncture, median (IQR), minutes (63 missing)	265 (185–385)	232.5 (153.75–636.25)	265 (185–385)	280 (195–385)	0.66
Intravenous thrombolysis, *n* (%)	84 (40%)	5 (21.7)	18 (36.0)	61 (44.5)	0.095

**Table 2:** Procedural and outcome characteristics.

	All Patients	mTICI 0-2a	mTICI 2b	mTICI 3	*p*=
Anesthesia, *n* (%), (108 missing)					
Local anesthesia	0 (0)	0 (0)	0 (0)	0 (0)	0.12
Conscious sedation	1 (1.0)	0 (0)	1 (4.5)	0 (0)	
Primary general anesthesia	100 (98.0)	16 (100)	20 (90.9)	64 (100)	
Secondary general anesthesia	1 (1.0)	0 (0)	1 (4.5)	0 (0)	
Interventional duration min, median (IQR), (1 missing)	60 (31–105)	83 (50–146)	80 (48–124.5)	45 (25.5–95)	<0.001
Reperfusion attempts, median (IQR)	2 (1–3)	1 (0–2)	2 (1–4)	1 (1–2)	0.008
Basilar stenting, *n* (%)	47 (22.4)	2 (8.7)	14 (28)	31 (22.6)	0.18
Vertebral artery stenting, *n* (%)	17 (8.1)	2 (8.7)	7 (14.0)	8 (5.8)	0.19
Sole aspiration, *n* (%)	63 (30.6)	3 (13.6)	11 (22.9)	49 (36.9)	0.045
**Outcome Characteristics**					
Post-treatment NIHSS, median (IQR), (16 missing)	10 (2–42)	25 (5–42)	11 (4–42)	7.5 (2–17.75)	0.005
Substantial neurological improvement, *n* (%), (14 missing)	69 (35.2)	4 (19.0)	10 (21.3)	55 (43.0)	0.008
post-treatment mRS, median (IQR), (11 missing)	4 (2–6)	5 (4–6)	5 (3–6)	4 (1–5)	0.001
mRS 0–3 (good clinical outcome), *n* (%), (11 missing)	74 (37.2)	4 (18.2)	13 (26.5)	57 (44.5)	0.013
mRS >3, *n* (%)	125 (62.8)	18 (81.8)	36 (73.5)	71 (55.5)	
mRS after 3 months, median (IQR), (125 missing)	4 (1–6)	6 (3–6)	4 (1–6)	3 (1–6)	0.09
**Safety**					
Symptomatic ICH, *n* (%)	6 (2.9)	0 (0)	0 (0)	6 (4.4)	0.19
In-house Mortality, *n* (%), (9 missing)	50 (24.9)	9 (39.1)	18 (36.7)	23 (17.8)	0.008

**Table 3:** Multivariate logistic regression to predict a poor outcome (post-treatment mRS of 5 or 6).

Variable	OR	Lower 95% CI	Upper 95% CI	*p*=
Age	1.04	1.01	1.08	0.0138
Underlying BA Stenosis	4.86	2.15	10.98	0.0001
Admission NHISS	1.09	1.04	1.13	0.0001
mTICI	1.89	1.09	3.25	0.0224

## Data Availability

No publicly archived datasets were analyzed or generated during the study.

## Supplementary Materials

The following supporting information can be downloaded at: https://www.mdpi.com/article/10.3390/diagnostics14212348/s1, Figure S1: Rates of successful recanalization based on BAO etiology (BAO due to BS [red] vs. embolic BAO [blue], *p* = 0.04); Table S1: Patient characteristics and factors associated with poor clinical outcome; Figure S2: Forest-Plot showing results of multivariate logistic regression to predict a poor clinical outcome (mRS 5-6).

## Abbreviations

BA	Basilar artery
BAO	Basilar artery occlusion
BS	Basilar stenosis
EVT	Endovascular therapy
IVT	Intravenous thrombolysis
mRS	Modified Rankin Scale
mTICI	Modified Thrombolysis in Cerebral Infarction
NIHSS	National Institutes of Health Stroke Scale
RCT	Randomized-controlled trial
