# Supplementary material for: Basilar Stenosis Reduces the Impact of Successful Recanalization on Outcome in Basilar Artery Occlusion
Source: Diagnostics (Basel). 2024 Oct 22;14(21):2348. doi: 10.3390/diagnostics14212348 (PMC11544911; doi:10.3390/diagnostics14212348)
Supplement: Supplementary file 1 [file diagnostics-14-02348-s001.zip › diagnostics-3234949-supplementary.pdf]

Supplemental Material

Table of contents Page

Figure S1	2
Table S1	3
Figure S2	4

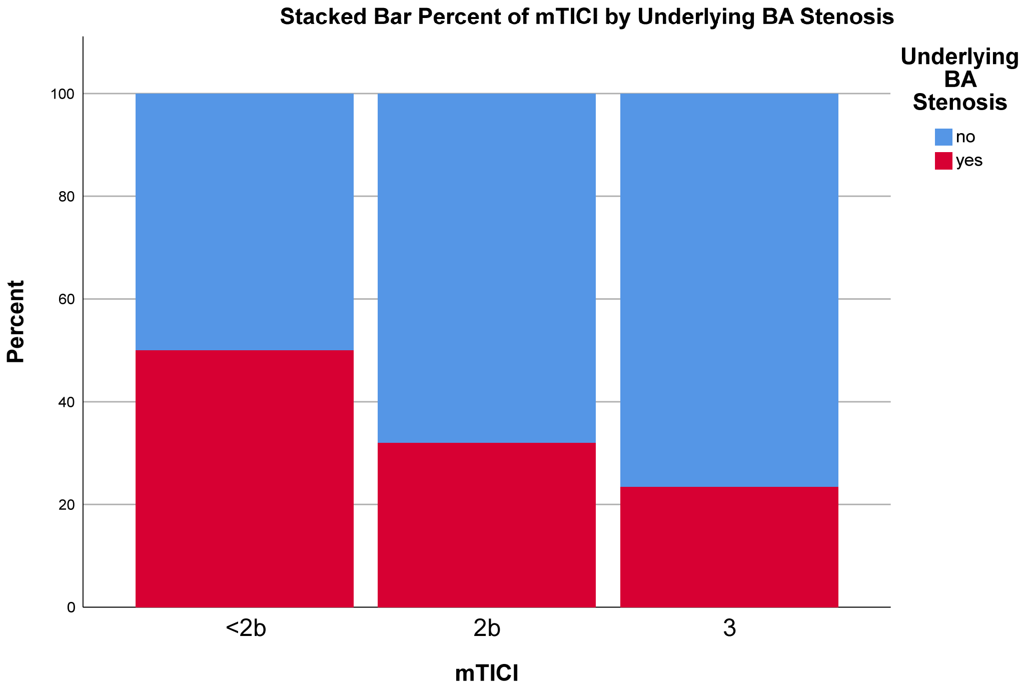

Figure S1. Rates of successful recanalization based on BAO etiology (BAO due to BS [red] vs. embolic BAO [blue], p=0.04).

	Values (n, percentage for categorical variables; median/IQR for parametric variables)	Univariate analysis (p-values)
**Baseline characteristics**		
Age, median (IQR)	74 (63 - 81)	**0.034**
Male sex, n (%)	119 (59.8%)	0.389
NIHSS at admission, median (IQR)	13 (6.5 - 22)	**<0.001**
Pre-stroke mRS, median (IQR)	0 (0 - 0.5)	0.443
Underlying basilar stenosis, n (%)	56 (28.6%)	**<0.001**
Peri-interventional intravenous tPA, n (%)	81 (40.7%)	0.247
**Risk Factors**		
Diabetes Mellitus, n (%)	32 (16.5%)	**<0.001**
Atrial fibrillation, n (%)	78 (39.8%)	0.057
Hypertension, n (%)	142 (71.7%)	0.269
Previous TIA or Stroke, n (%)	45 (23.0%)	0.496
Dyslipidemia, n (%)	38 (19.1%)	0.470
**Procedural variables**		
mTICI (3, 2b, below 2b), n (%)	3: 137 (65.2.4%); 2b: 50 (23.8%); below 2b: 23 (11.0%)	**0.026**
Reperfusion attempts, median (IQR)	2 (1 - 3)	**0.002**
Interventional duration, min, median (IQR)	60 (31 - 105)	**<0.001**
Time from symptom onset to groin puncture, min, median (IQR)	265 (185 - 385)	0.993
Sole Aspiration, n (%)	59 (30.3%)	**0.005**
Basilar stenting, n (%)	45 (22.6%)	**<0.001**
Vertebral artery stenting, n (%)	17 (8.5%)	0.617

Table S1. Patient characteristics and factors associated with poor clinical outcome.

Baseline demographic, clinical, and interventional data were collected for all patients, and univariate analyses were conducted to assess differences between groups in the occurrence of poor clinical outcomes (mRS 5-6). The Mann-Whitney-U test was used for comparing metric or ordinal data, Fisher's exact test for dichotomous categorical variables, and the Pearson Chi-Square test for categorical variables with three groups (TICI score). Abbreviations: mRS - modified Rankin Scale, NIHSS - National Institute of Health Stroke Scale, TICI - modified thrombolysis in cerebral infarction, tPA - tissue Plasminogen Activator, IQR - interquartile range. The number of missing cases was subtracted from the total number of cases for each variable to calculate percentage for categorical variables.

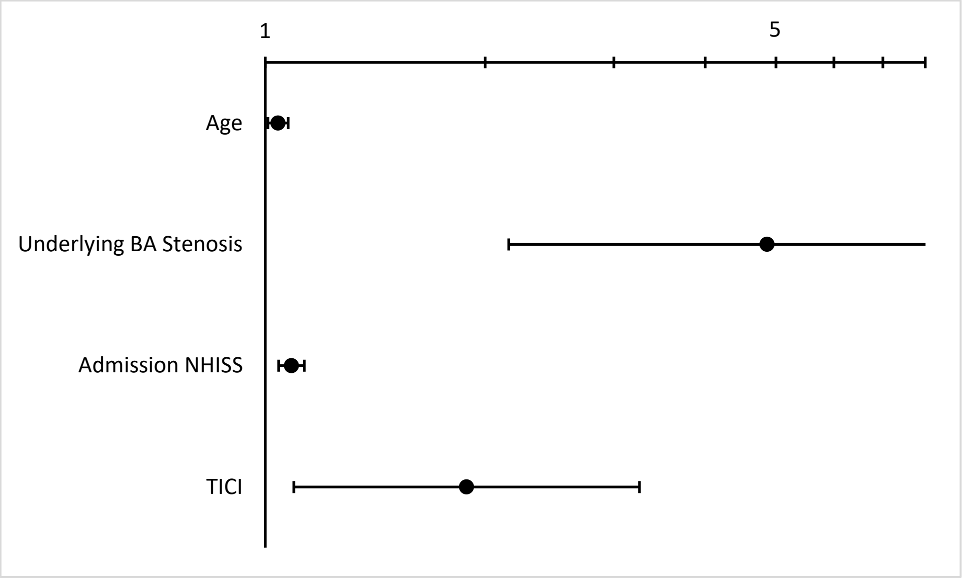

Figure S2. Forest-Plot showing results of multivariate logistic regression to predict a poor clinical outcome (mRS 5-6).
